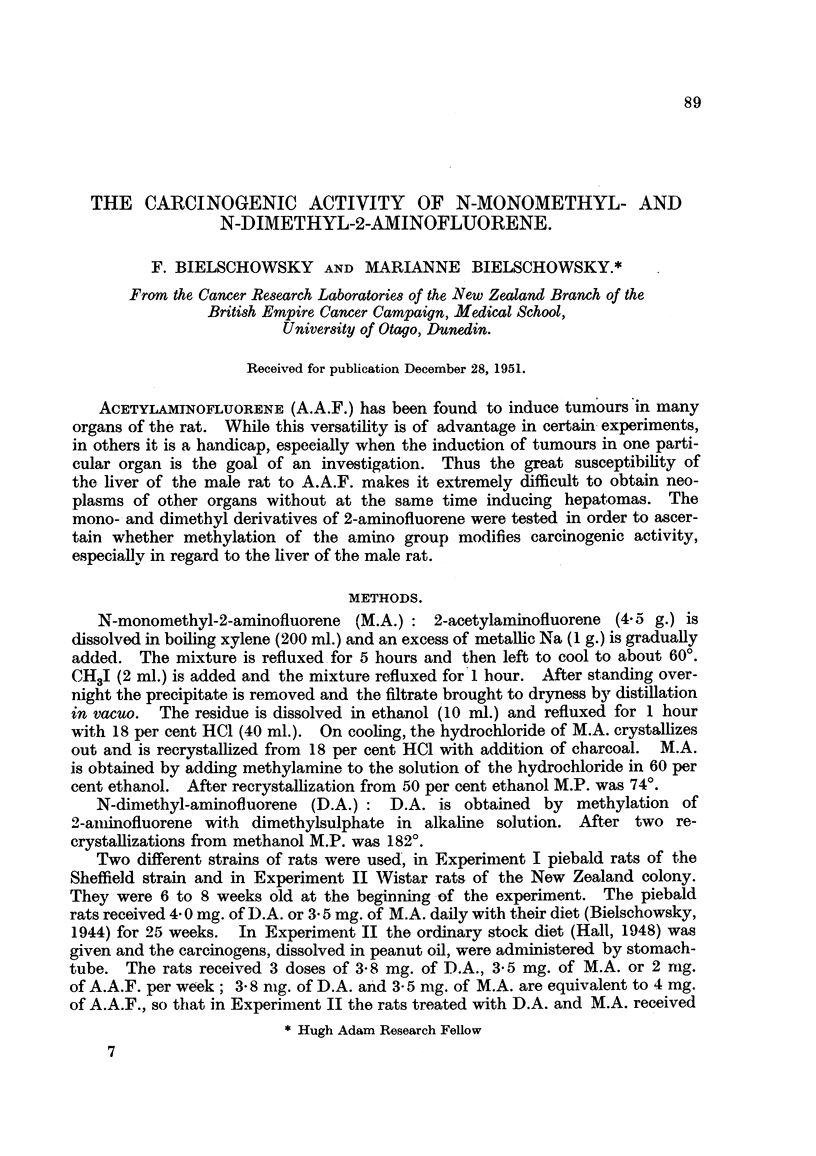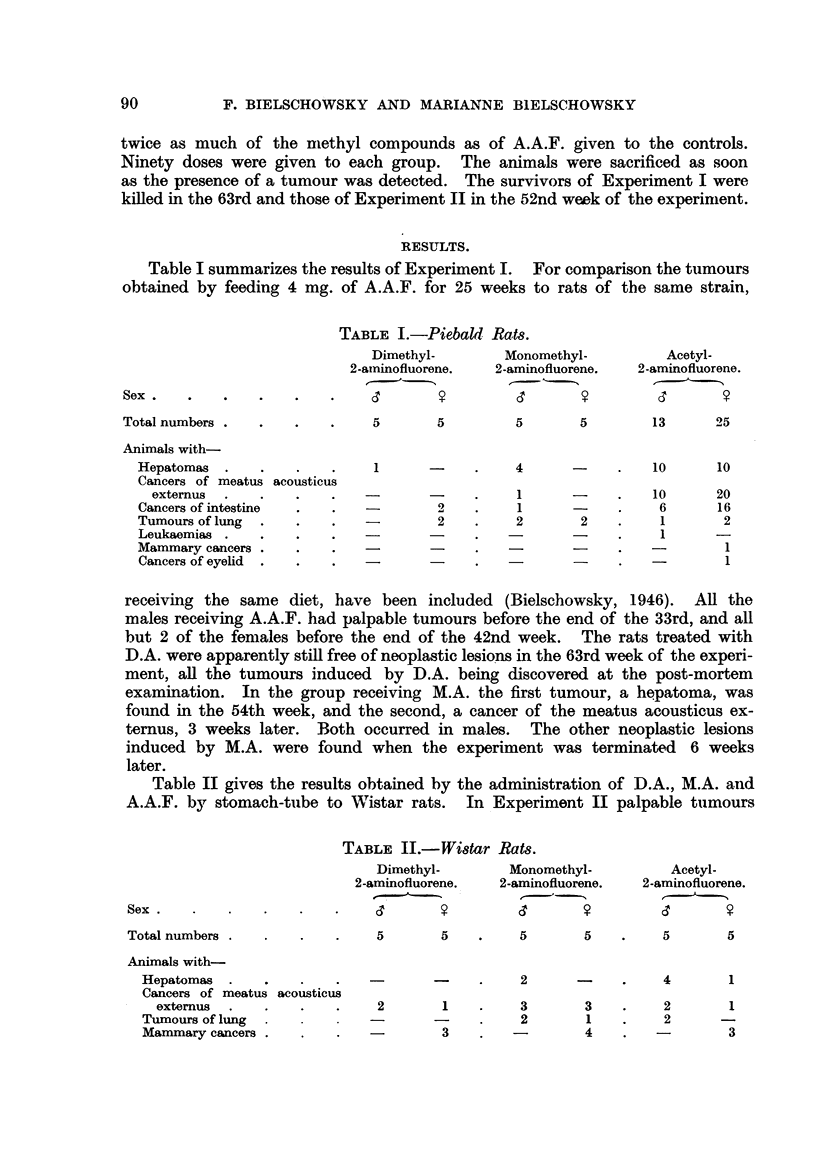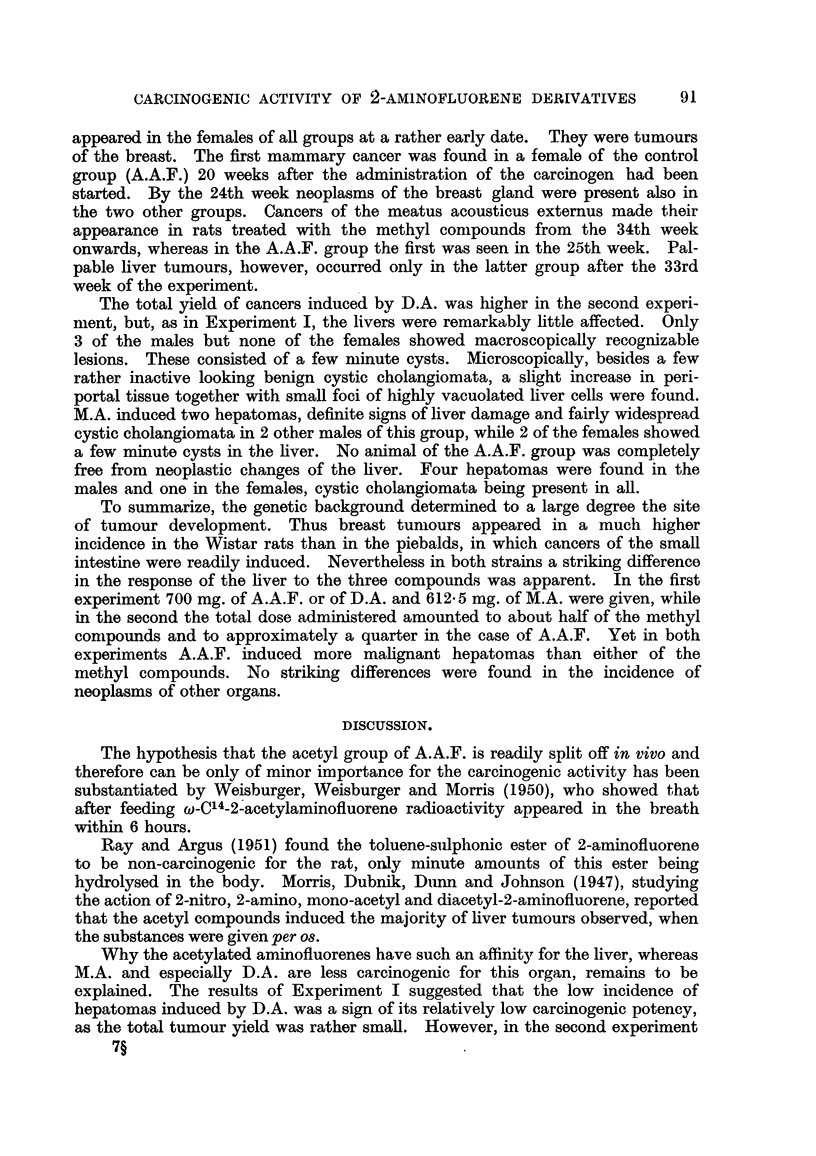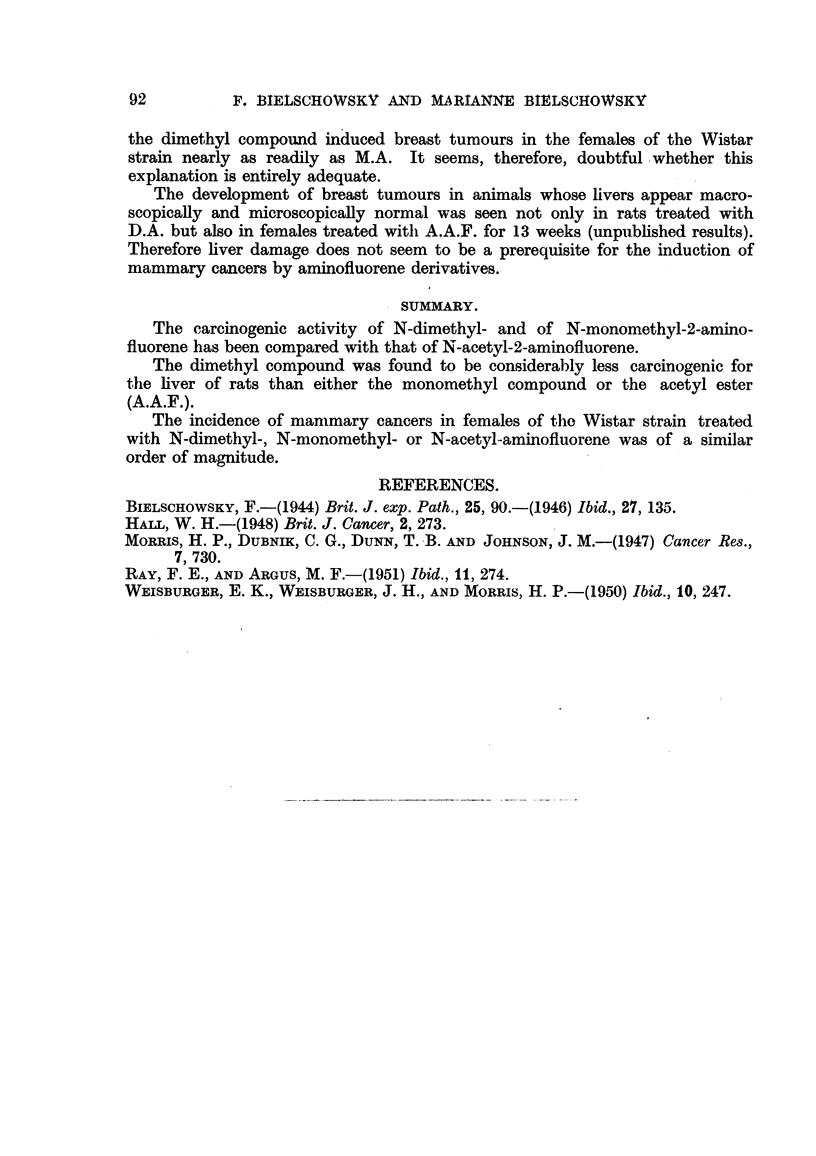# The Carcinogenic Activity of N-Monomethyl- and N-Dimethyl-2-aminofluorene

**DOI:** 10.1038/bjc.1952.9

**Published:** 1952-03

**Authors:** F. Bielschowsky, Marianne Bielschowsky


### THE CARCINOGENIC ACTIVITY OF N-MONOMETHYL- AND N-DIMETHYL-2-AMINOFLUORENE.

F. BIELSCHOWSKY AND MARIANNE BIELSCHOWSKY.*

*From the Cancer Research Laboratories of the New Zealand Branch of the British Empire Cancer Campaign, Medical School, University of Otago, Dunedin.*

Received for publication December 28, 1951.

ACETYLAMINOFLUORENE (A.A.F.) has been found to induce tumours in many organs of the rat. While this versatility is of advantage in certain experiments, in others it is a handicap, especially when the induction of tumours in one particular organ is the goal of an investigation. Thus the great susceptibility of the liver of the male rat to A.A.F. makes it extremely difficult to obtain neoplasms of other organs without at the same time inducing hepatomas. The mono- and dimethyl derivatives of 2-aminofluorene were tested in order to ascertain whether methylation of the amino group modifies carcinogenic activity, especially in regard to the liver of the male rat.

### METHODS.

N-monomethyl-2-aminofluorene (M.A.) : 2-acetylaminofluorene (4·5 g.) is dissolved in boiling xylene (200 ml.) and an excess of metallic Na (1 g.) is gradually added. The mixture is refluxed for 5 hours and then left to cool to about 60°. $CH_3I$ (2 ml.) is added and the mixture refluxed for 1 hour. After standing overnight the precipitate is removed and the filtrate brought to dryness by distillation *in vacuo*. The residue is dissolved in ethanol (10 ml.) and refluxed for 1 hour with 18 per cent HCl (40 ml.). On cooling, the hydrochloride of M.A. crystallizes out and is recrystallized from 18 per cent HCl with addition of charcoal. M.A. is obtained by adding methylamine to the solution of the hydrochloride in 60 per cent ethanol. After recrystallization from 50 per cent ethanol M.P. was 74°.

N-dimethyl-aminofluorene (D.A.) : D.A. is obtained by methylation of 2-aminofluorene with dimethylsulphate in alkaline solution. After two recrystallizations from methanol M.P. was 182°.

Two different strains of rats were used, in Experiment I piebald rats of the Sheffield strain and in Experiment II Wistar rats of the New Zealand colony. They were 6 to 8 weeks old at the beginning of the experiment. The piebald rats received 4·0 mg. of D.A. or 3·5 mg. of M.A. daily with their diet (Bielschowsky, 1944) for 25 weeks. In Experiment II the ordinary stock diet (Hall, 1948) was given and the carcinogens, dissolved in peanut oil, were administered by stomach-tube. The rats received 3 doses of 3·8 mg. of D.A., 3·5 mg. of M.A. or 2 mg. of A.A.F. per week ; 3·8 mg. of D.A. and 3·5 mg. of M.A. are equivalent to 4 mg. of A.A.F., so that in Experiment II the rats treated with D.A. and M.A. received

* Hugh Adam Research Fellow twice as much of the methyl compounds as of A.A.F. given to the controls. Ninety doses were given to each group. The animals were sacrificed as soon as the presence of a tumour was detected. The survivors of Experiment I were killed in the 63rd and those of Experiment II in the 52nd week of the experiment.

### RESULTS.

Table I summarizes the results of Experiment I. For comparison the tumours obtained by feeding 4 mg. of A.A.F. for 25 weeks to rats of the same strain,

TABLE I.—*Piebald Rats.*

| | Dimethyl-2-aminofluorene. | | Monomethyl-2-aminofluorene. | | Acetyl-2-aminofluorene. | |
|---|---|---|---|---|---|---|
| Sex | ♂ | ♀ | ♂ | ♀ | ♂ | ♀ |
| Total numbers | 5 | 5 | 5 | 5 | 13 | 25 |
| Animals with— | | | | | | |
| Hepatomas | 1 | — | 4 | — | 10 | 10 |
| Cancers of meatus acousticus externus | — | — | 1 | — | 10 | 20 |
| Cancers of intestine | — | 2 | 1 | — | 6 | 16 |
| Tumours of lung | — | 2 | 2 | 2 | 1 | 2 |
| Leukaemias | — | — | — | — | 1 | — |
| Mammary cancers | — | — | — | — | — | 1 |
| Cancers of eyelid | — | — | — | — | — | 1 |

receiving the same diet, have been included (Bielschowsky, 1946). All the males receiving A.A.F. had palpable tumours before the end of the 33rd, and all but 2 of the females before the end of the 42nd week. The rats treated with D.A. were apparently still free of neoplastic lesions in the 63rd week of the experiment, all the tumours induced by D.A. being discovered at the post-mortem examination. In the group receiving M.A. the first tumour, a hepatoma, was found in the 54th week, and the second, a cancer of the meatus acousticus externus, 3 weeks later. Both occurred in males. The other neoplastic lesions induced by M.A. were found when the experiment was terminated 6 weeks later.

Table II gives the results obtained by the administration of D.A., M.A. and A.A.F. by stomach-tube to Wistar rats. In Experiment II palpable tumours

TABLE II.—*Wistar Rats.*

| | Dimethyl-2-aminofluorene. | | Monomethyl-2-aminofluorene. | | Acetyl-2-aminofluorene. | |
|---|---|---|---|---|---|---|
| Sex | ♂ | ♀ | ♂ | ♀ | ♂ | ♀ |
| Total numbers | 5 | 5 | 5 | 5 | 5 | 5 |
| Animals with— | | | | | | |
| Hepatomas | — | — | 2 | — | 4 | 1 |
| Cancers of meatus acousticus externus | 2 | 1 | 3 | 3 | 2 | 1 |
| Tumours of lung | — | — | 2 | 1 | 2 | — |
| Mammary cancers | — | 3 | — | 4 | — | 3 |

appeared in the females of all groups at a rather early date. They were tumours of the breast. The first mammary cancer was found in a female of the control group (A.A.F.) 20 weeks after the administration of the carcinogen had been started. By the 24th week neoplasms of the breast gland were present also in the two other groups. Cancers of the meatus acousticus externus made their appearance in rats treated with the methyl compounds from the 34th week onwards, whereas in the A.A.F. group the first was seen in the 25th week. Palpable liver tumours, however, occurred only in the latter group after the 33rd week of the experiment.

The total yield of cancers induced by D.A. was higher in the second experiment, but, as in Experiment I, the livers were remarkably little affected. Only 3 of the males but none of the females showed macroscopically recognizable lesions. These consisted of a few minute cysts. Microscopically, besides a few rather inactive looking benign cystic cholangiomata, a slight increase in periportal tissue together with small foci of highly vacuolated liver cells were found. M.A. induced two hepatomas, definite signs of liver damage and fairly widespread cystic cholangiomata in 2 other males of this group, while 2 of the females showed a few minute cysts in the liver. No animal of the A.A.F. group was completely free from neoplastic changes of the liver. Four hepatomas were found in the males and one in the females, cystic cholangiomata being present in all.

To summarize, the genetic background determined to a large degree the site of tumour development. Thus breast tumours appeared in a much higher incidence in the Wistar rats than in the piebalds, in which cancers of the small intestine were readily induced. Nevertheless in both strains a striking difference in the response of the liver to the three compounds was apparent. In the first experiment 700 mg. of A.A.F. or of D.A. and 612·5 mg. of M.A. were given, while in the second the total dose administered amounted to about half of the methyl compounds and to approximately a quarter in the case of A.A.F. Yet in both experiments A.A.F. induced more malignant hepatomas than either of the methyl compounds. No striking differences were found in the incidence of neoplasms of other organs.

### DISCUSSION.

The hypothesis that the acetyl group of A.A.F. is readily split off *in vivo* and therefore can be only of minor importance for the carcinogenic activity has been substantiated by Weisburger, Weisburger and Morris (1950), who showed that after feeding $\omega$-$C^{14}$-2-acetylaminofluorene radioactivity appeared in the breath within 6 hours.

Ray and Argus (1951) found the toluene-sulphonic ester of 2-aminofluorene to be non-carcinogenic for the rat, only minute amounts of this ester being hydrolysed in the body. Morris, Dubnik, Dunn and Johnson (1947), studying the action of 2-nitro, 2-amino, mono-acetyl and diacetyl-2-aminofluorene, reported that the acetyl compounds induced the majority of liver tumours observed, when the substances were given *per os*.

Why the acetylated aminofluorenes have such an affinity for the liver, whereas M.A. and especially D.A. are less carcinogenic for this organ, remains to be explained. The results of Experiment I suggested that the low incidence of hepatomas induced by D.A. was a sign of its relatively low carcinogenic potency, as the total tumour yield was rather small. However, in the second experiment the dimethyl compound induced breast tumours in the females of the Wistar strain nearly as readily as M.A. It seems, therefore, doubtful whether this explanation is entirely adequate.

The development of breast tumours in animals whose livers appear macroscopically and microscopically normal was seen not only in rats treated with D.A. but also in females treated with A.A.F. for 13 weeks (unpublished results). Therefore liver damage does not seem to be a prerequisite for the induction of mammary cancers by aminofluorene derivatives.

SUMMARY.

The carcinogenic activity of N-dimethyl- and of N-monomethyl-2-aminofluorene has been compared with that of N-acetyl-2-aminofluorene.

The dimethyl compound was found to be considerably less carcinogenic for the liver of rats than either the monomethyl compound or the acetyl ester (A.A.F.).

The incidence of mammary cancers in females of the Wistar strain treated with N-dimethyl-, N-monomethyl- or N-acetyl-aminofluorene was of a similar order of magnitude.

### REFERENCES.

Bielschowsky, F.—(1944) *Brit. J. exp. Path.*, **25**, 90.—(1946) *Ibid.*, **27**, 135.
Hall, W. H.—(1948) *Brit. J. Cancer*, **2**, 273.
Morris, H. P., Dubnik, C. G., Dunn, T. B. and Johnson, J. M.—(1947) *Cancer Res.*, **7**, 730.
Ray, F. E., and Argus, M. F.—(1951) *Ibid.*, **11**, 274.
Weisburger, E. K., Weisburger, J. H., and Morris, H. P.—(1950) *Ibid.*, **10**, 247.